# Remodeling of hepatic stellate cells orchestrated the stroma-derived oxaliplatin-resistance through CCN3 paracrine in hepatocellular carcinoma

**DOI:** 10.1186/s12885-019-6362-1

**Published:** 2019-12-05

**Authors:** Xia Liao, Yang Bu, Fan Chang, Fengan Jia, Ge Song, Xuelian Xiao, Mei Zhang, Pengbo Ning, Qingan Jia

**Affiliations:** 1 Department of Nutrition, First Affiliated Hospital of Xi’an Jiaotong University, Xi’an, 710061 China; 2 Department of Hepatobiliary Surgery, General Hospital, Ningxia Medical University, Yinchuan, 750001 China; 3 Metabolite Research Center, Shaanxi Institute of Microbiology, Xi’an, 710043 China; 4 Department of Hepatobiliary Surgery, First Affiliated Hospital of Xi’an Jiaotong University, 277 West Yanta Road, Xi’an, 710061 China; 5 School of Life Science and Technology, Xidian University, Xi’an, Shaanxi China

**Keywords:** Hepatocellular carcinoma, Microenvironment, Oxaliplatin resistance, CCN3, Hepatic stellate cells

## Abstract

**Background:**

Hepatic stellate cells (HSCs) have a key role in fibrogenesis and in the filtrates of the hepatocellular carcinoma (HCC) stroma, in which they are remodeled and play a critical role in HCC progression. However, the precise role of HSCs trending, infiltration and paracrine in orchestrating the stroma-derived oxaliplatin-resistance in HCC is still vague.

**Methods:**

The chemo-resistant models were established to explore the correlation between HSC cells and the condition of chemoresistance. The HCC clinical samples were collected to confirm this phenomenon. Then, the relationship between secretory CCN3 from oxaliplatin-resistant HCC and the infiltration of HSCs in associated HCC microenvironment was evaluated. Finally, the role and mechanism of HSCs remodeling in the orchestration of oxaliplatin-resistant HCC were explored.

**Results:**

The increased infiltration of HSCs and collagen accumulation were found in the microenvironment of oxaliplatin-resistant HCC. The cDNA profiles of the oxaliplatin-resistant HCC was reanalyzed, and CCN3 was one of the significantly increased genes. In HCC clinical samples, the levels of CCN3 and α-SMA are positively correlated, and high expression of CCN3 and α-SMA are positively associated with malignant phenotype and poor prognosis. Then the enhanced abilities of migration and proliferation of HSCs, and elevation of the cytokines paracrine from HSCs relating to HCC malignancy were proved in vitro and in vivo, and which were related to CCN3-ERK signaling pathway activation.

**Conclusions:**

HSCs remodeling are positively related to CCN3 paracrine in hepatocellular carcinoma, which orchestrated the stroma-derived resistance to chemotherapy in HCC.

## Background

Primary liver cancer is the second leading cause of cancer death worldwide, with China alone accounting for about 50% of the total number of cases and deaths, and 80% primary liver cancers occurring worldwide are hepatocellular carcinoma (HCC) [1]. HCC occurs in a large percentage of cases during the clinical course of chronic infection by hepatitis B virus and hepatitis C virus leading to cirrhosis. Hepatic stellate cells (HSCs) have a key role in fibrogenesis and in filtrates of the HCC stroma, and enhance HCC malignant progression. While, the precise role of HSCs remodeling is still vague in stroma-derived chemoresistance [2].

CCN3 (Nephroblastoma Overexpressed proteins, NOV) is one of the six-member family of cysteine-rich secretory proteins found in humans that emerged as localized multitasking signal integrators in the microenvironment, and play an important role in modifying the cellular phenotype [3]. The most ubiquitous function of the CCN3 is its ability to orchestrate the inflammatory microenvironment [4]. Previously, by cDNA microarrays, we found oxaliplatin-resistant HCC exhibited the increased expression of CCN3 [5]. Thus far, the expression of CCN3 in HCC, and the precise physiological function and mechanism of action of CCN3 remain elusive. So, it is important to explore the role and mechanism of CCN3 paracrine in the remodeling of HSCs in HCC microenvironment.

Through collecting HCC clinical samples and establishing oxaliplatin-resistant models, we explored the relationship between secretory CCN3 from HCC and the infiltration of HSCs and collagen accumulation in HCC microenvironment. We also evaluated the associated mechanism of HSC infiltration and remodeling in HCC with high expression of CCN3. In this study, our findings suggested that CCN3 paracrined by HCCs play an important role in the HSCs-derived oxaliplatin-resistance, which may be used as a potential therapeutic target.

## Methods

### Patients and follow-up

For this study, 86 paired HCC samples were used for immunohistochemistry. The samples were obtained from patients who underwent curative resection between January 2004 and December 2006 at the Liver Cancer Institute and Zhongshan Hospital of Fudan University. Curative resection was defined as the complete resection of tumor nodules, leaving the tumor margins free of cancer upon histologic examination. Histopathologic diagnosis was performed according to the WHO criteria. Patients were followed-up after the surgical treatment until December 2013. The median follow-up period was 63 months (range, 0–110 months). The clinicopathologic characteristics of all HCC patients in this study are provided in Table 1. Another 98 HCC samples were used for real-time polymerase chain reaction (PCR) analysis for evaluating the relationship of CCN3 and α-SMA in mRNA level, and 373 HCC patients with or without cirrhosis were used for survival statistical analysis. Histopathologic diagnosis was performed according to the WHO criteria. Written consent was obtained from patients who received curative resection at the Liver Cancer Institute of Zhongshan Hospital of Fudan University, and ethical approval was obtained from the Research Ethics Committee of Fudan University (Permit Number:2015–138).

**Table 1:** Correlations between CCN3/SMA and clinicopathology feature in 86 patients with HCC

Variable	No. of Patient			No. of Patient		
	CCN3^high^	CCN3^low^	*p*	SMA ^high^	SMA ^low^	*p*
Age, y						
≥ 53	14	25	0.419	18	21	0.397
<53	19	28		26	21	
Sex						
Men	26	44	0.624	36	34	0.918
Women	7	9		8	8	
HBsAg						
Positive	30	48	0.958^*^	39	39	0.501^*^
Negative	3	5		5	3	
Cirrhosis						
Yes	29	46	0.883^*^	42	34	0.085^*^
No	4	7		2	9	
Serum, AFP						
≥ 20	21	32	0.762	29	24	0.403
<20	12	21		15	18	
Serum, ALT						
≥ 75	11	27	0.110	15	23	0.053
<75	22	26		29	19	
Tumor dimension						
≥ 5 cm	9	4	**0.013**	10	3	0.044^*^
<5 cm	24	49		34	39	
No. of tumors						
Multiple	4	2	0.139^*^	3	3	0.953^*^
Single	29	51		41	39	
Vascular invasion						
Yes	9	4	0.023^*^	7	6	0.834
No	24	49		37	36	
Tumor encapsulation						
Complete	8	27	**0.014**	12	23	**0.009**
None	25	26		32	19	

For CCN3, and SMA median values were used as cut-off points for definition of subgroups (low expression and high expression groups)

^*^Fisher’s exact tests, and Chi-square tests for all other analyses

Boldface in table indicates the difference has statistical significance (*p*<0.05)

### Vector construction, transfection, and Lentivirus transduction

Human full-length CCN3 cDNA (NM_002165) was obtained from GeneCards (Shanghai, China) and cloned into the pCDH lentiviral expression vector (System Biosciences, CA, USA). The amplified fragment was inserted into the pCDH plasmid (between *Xba*I and *Eco*RI sites) by using the In-Fusion® HD Cloning Kit (Takara, Tokyo, Japan). The target sequences of lentiviral shRNA expression plasmids PLKO.1 are as follows: CCCACCATCAAAGGAATATAA (Sh1), CGCACCAAGAAGTCACTCAAA (Sh2), and CACCAATAGGAACCGTCAATG (Sh3).

### Cell lines and animals

HCC cell lines MHCC97H (*Homo sapiens*, RRID: CVCL_4972), Hep3B (*Homo sapiens*, RRID: CVCL 0326), and hepatic stellate cell lines LX2 (*Homo sapiens*, RRID: CVCL_5792) were obtained from the Liver Cancer Institute of Fudan University (Shanghai, China). All cells were maintained in Dulbecco’s Modified Eagle’s Medium (DMEM; GICBO, Grand Island, NY), supplemented with 10% fetal bovine serum (FBS; GICBO) at 37 °C in a humidified incubator with 5% CO_2_. Cells were routinely screened for the presence of mycoplasma (Mycoplasma Detection Kit, Roche Diagnostics). Oxaliplatin-resistant MHCC97H cell line was constructed and named MHCC97H-OXA as previously described [5]. It was routinely screened for presence of mycoplasma (Mycoplasma Detection Kit, Roche Diagnostics) during the study period.

A total of 24 male BALB/c nu/nu mice (aged 4–6 weeks and weighing approximately 20 g) were obtained from the Chinese Academy of Science (SLRC, Shanghai, China) and raised in a controlled environment with 25 °C under standard pathogen-free conditions and a natural light/dark cycle (morning 8:00; afternoon 8:00), and were provided with water and standard diet. To produce tumors, MHCC97H cells were injected into the upper right flank region of 12 mice. Seven days later, half of the mice were treated with 0.1 ml oxaliplatin (10 mg/kg) via tail vein injection once a week, and the other half were similarly injected with 0.1 ml 5% glucose solution (GS) as a control as previously described [5]. We also performed subcutaneous injections of MHCC97H-CCN3-sh and MHCC97H-Mock into the upper right flank region of total 6 mice. MHCC97H-CCN3 and MHCC97H-Vector cells were injected into the upper left flank region of total 6 mice. Four weeks later, orthotopic xenografts were measured, and which were used for immunohistochemistry. Intraperitoneal injection of pentobarbital (5 mg/kg) combined with cervical dislocation was used for the killing of mice after the study. The study protocol was approved by the Shanghai Medical Experimental Animal Care Commission (Permit Number:2016–120).

### RNA extraction and qRT-PCR

Total RNA was extracted from HCC cells using TRIzol® reagent (Invitrogen, Carlsbad, CA, USA). The primers used for the amplification of human genes are the following: CCN3, 5′-CACGGCGGTAGAGGGAGATAA-3′ (forward) and 5′-TGGGCCACAGATCCACTTTTC-3′ (reverse); α-SMA, 5′-TCCCTTGAGAAGAGTTACGAGTT-3′ (forward) and 5′-CATGATGCTGTTGTAGGTGGTT-3′ (reverse).

### Cell migration ability assays

LX2 cells were cultured in different conditioned media, and cell migration was performed as previously described [6]. Migration was assessed by transwell assays using Boyden chambers (Corning, Tewksbury, MA, USA) using the Conditioned Medium (CM) of MHCC97H-Con-CM, and MHCC97H-Oxa-CM. Then, 5 × 10^4^ cells in serum free DMEM were seeded into the upper chamber of each well on the membrane (8.0 μm pore size) of a 24-well plate. CM was added to the lower chamber of each well. After 48 h, cells reaching the underside of the membrane were stained with Giemsa (Sigma-Aldrich) and counted at × 200 magnification.

### Immunohistochemistry, immunofluorescence, Immunoblotting and ELISA assays

Immunohistochemistry, Immunofluorescence, Immunoblotting and ELISA were performed as previously described [7]. The BCA Protein Assay Kit (Beyotime, Shanghai, China) was used to determine the concentration of extracted protein. Levels of CCN3 in the cultured supernatants were quantified by ELISA kits (R&D Lab Inc., Minneapolis, MN, USA). MEK1/2 inhibitor U0126, NFκB inhibitor EVP4593, and Sorafenib were obtained from Selleckchem (Houston, TX, USA). Primary antibodies used for immunofluorescence, immunoblotting and/or, immunohistochemistry were as follows: CCN3, α-SMA, p-C-RAF, ERK1/2, NFκB, TIMP-2, TGF-β, and p-ERK1/2 (Abcam, Cambridge, MA, USA), RANTES and C-RAF (Cell Signaling, Beverly, MA, USA), p-MEK (Epitomics, Burlingame, CA, USA), Actin (Jackson Labs, Bar Harbor, ME, USA).

### Cytokines antibody array

Cytokines microarrays were used to evaluate the changes in cytokine expression profiles of LX2 after CCN3 treatment. Total RNA extracted from CCN3 treated with LX2 and control cells were used for Cytokines antibody array analysis. Microarray analysis of three independent samples was performed according to the manufacturer’s instructions.

### Statistical analysis

Kaplan-Meier analysis was performed to compare CRR and OS between patients in different groups and statistic, and the values were generated by the Cox-Mantel log-rank test. Quantitative differences in the data on tumor volume, gene and protein expression levels, and cell number were evaluated by t-test. Statistical analyses were performed using SPSS 15.0 for Windows (SPSS) as previously described [8]. A *p*-value of less than 0.05 was considered statistically significant.

## Results

### Infiltration of fibroblast cells and collagen accumulation are increased in the microenvironment of oxaliplatin-resistant HCC

**Fig. 1:**
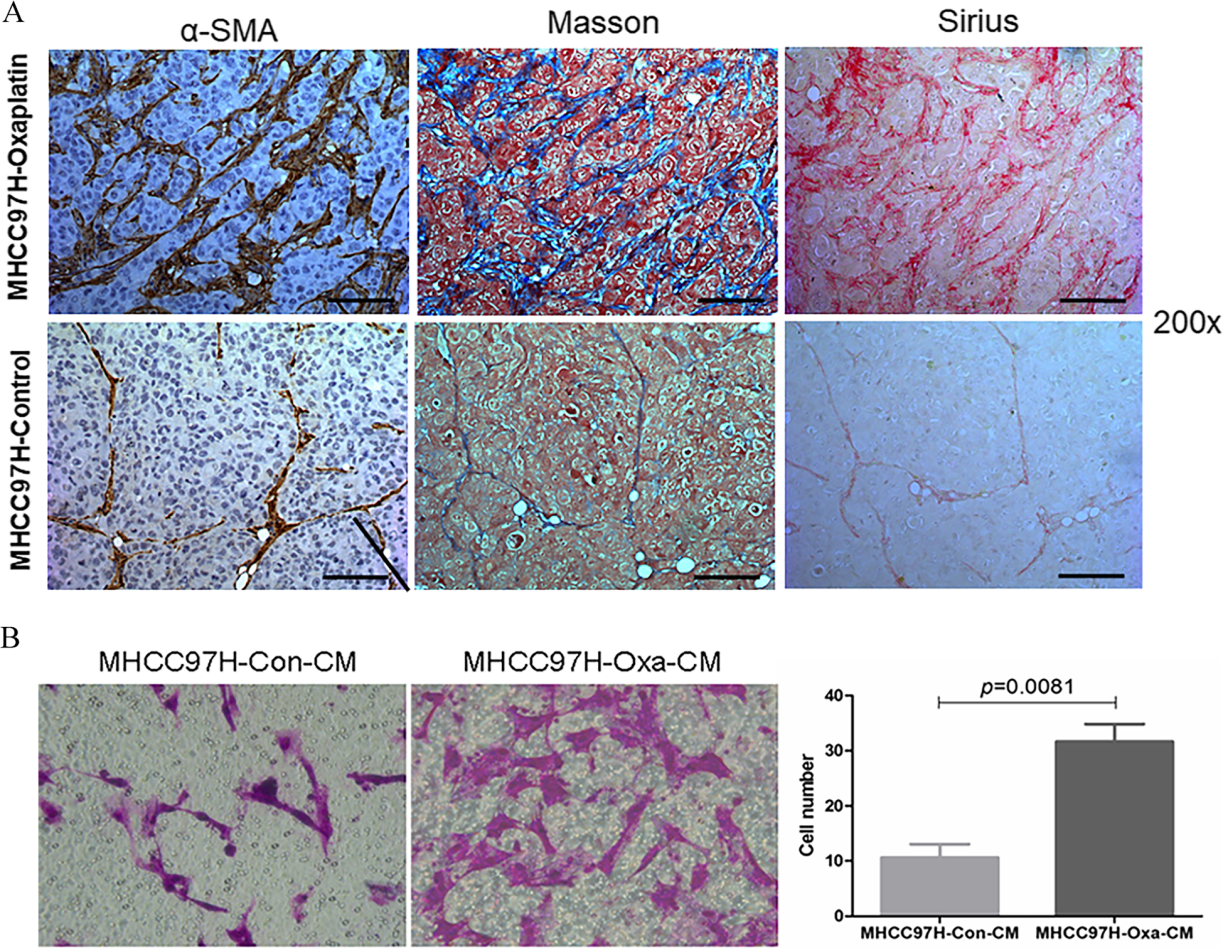
Infiltration of HSC increased in the microenvironment of oxaliplatin-resistant HCC. IHC. Masson and Sirius staining revealed the increased expression of fibrous connective tissue and collagen accumulation in oxaliplatin-resistant HCC tissue (**a**). The migration ability of LX2 was higher in the CM of oxaliplatin-resistant HCC than in the CM of wild type HCC (**b**)

To explore the mechanism of oxaliplatin resistance from the perspective of tumor microenvironment, immunohistochemical staining revealed the increased expression of α-SMA in HCC tissue from oxaliplatin-treated tumor mice in the same trend with the enhanced resistance to chemotherapy (Fig. 1a). Masson and Sirius staining also showed increased fibrous connective tissue and collagen accumulation in oxaliplatin-resistant HCC tissue (Fig. 1a). In vitro, we collected the CM of oxaliplatin-resistant HCC cell line MHCC97H-OXA and the associated wild type MHCC97H-CON, and proved the enhanced migration ability of LX2 in the CM of MHCC97H-OXA than in the CM of MHCC97H-CON (31.60 ± 7.16 vs. 10.60 ± 5.45 *p* = 0.0081) (Fig. 1b). In this section, we proved the number of fibroblast cells and collagen are accumulated in the microenvironment of oxaliplatin-resistant HCC.

### Expression of CCN3 is upregulated in oxaliplatin-resistant HCC

**Fig. 2:**
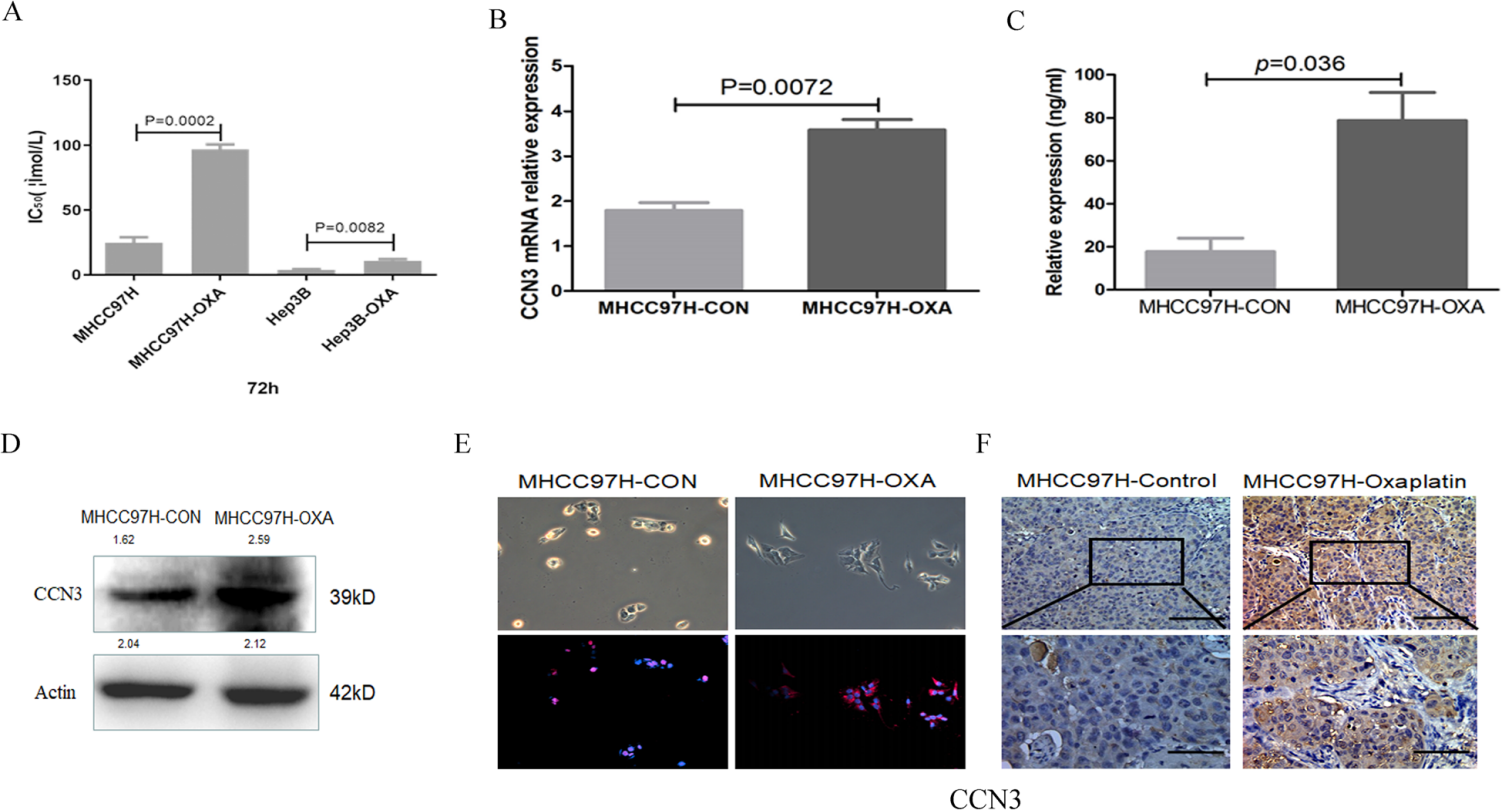
Expression of CCN3 is upregulated in oxaliplatin-resistant HCC. The increased resistance to oxaliplatin was in MHCC97H-OXA and Hep3B-OXA over that of parental cells (**a**). PCR (**b**), ELISA (**c**), Immunoblotting (**d**) and Immunofluorescence (**e**) revealed that the expression of CCN3 in MHCC97H-OXA cells was significantly higher than that of parental cells. IHC revealed CCN3 expression was significantly up-regulated in oxaliplatin-resistant HCC subcutaneous tissue (**f**)

Previously, we constructed oxaliplatin-resistant HCC models and found the expression profiles for 332 genes had 2-fold increase in the oxaliplatin-resistant HCC using cDNA microarrays [5]. We reanalyzed the expression profiles and found CCN3 was one of the significantly changed genes in oxaliplatin-resistant HCC. In the present study, the increased resistance was re-verified in MHCC97H-OXA (84.42 ± 8.26 mmol/l vs. 27.67 ± 5.37 mmol/l, *p* = 0.0002) and Hep3B-OXA (10.76 ± 2.36 mmol/l vs. 3.86 ± 0.68 mmol/l, *P* = 0.0082) cells to oxaliplatin over that of wild type cells with the higher concentration of IC_50_ (Fig. 2a). PCR (3.58 ± 0.52 vs. 1.79 ± 0.39, *p* = 0.0072, Fig. 2b) revealed that the gene expression of CCN3 in MHCC97H-OXA cells was increased significantly, compared with that of parental MHCC97H cells. ELISA (78.83 ± 25.76 ng/ml vs. 17.69 ± 12.65 ng/ml, *p* = 0.036, Fig. 2c), Immunoblotting (Fig. 2d) and immunofluorescence (Fig. *2*e) revealed that the expression of CCN3 in MHCC97H-OXA cells was significantly higher than that of the parental MHCC97H cells. In addition, immunohistochemical staining revealed the proportion of CCN3 expression was significantly up-regulated in oxaliplatin-resistant HCC subcutaneous tumor tissue (Fig. 2f). In this section, we proved expression of CCN3 is upregulated in oxaliplatin-resistant HCC, which may be the cause of increased infiltration of HSC after treated by oxaliplatin.

### The high expression of CCN3 and α-SMA are positively associated with malignant phenotype and poor prognosis in HCC clinical samples

**Fig. 3:**
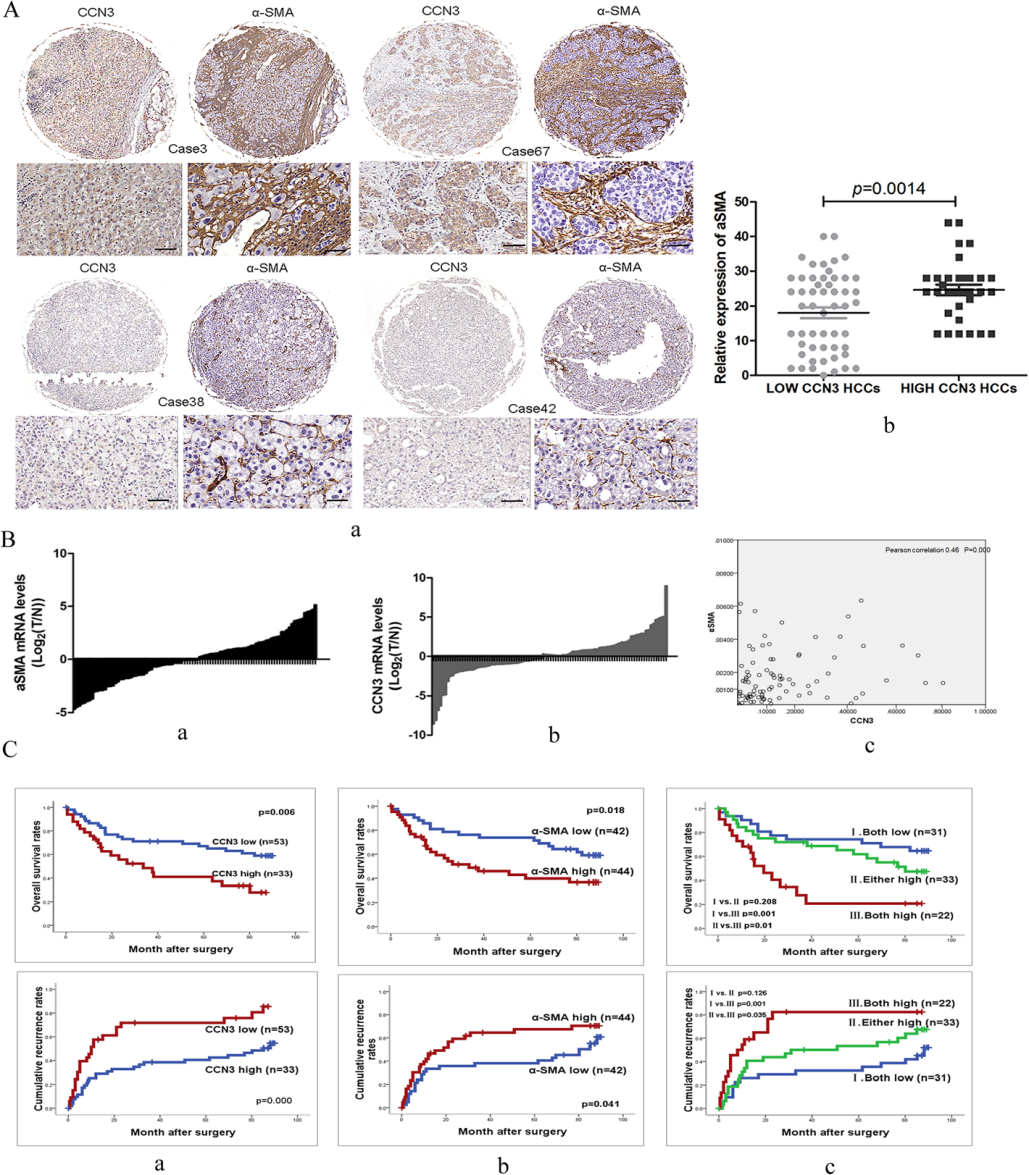
High expression of CCN3 and α-SMA is the positively associated poor prognosis in HCC. The expression of CCN3 is positively correlated with α-SMA (a, **A***;* a, b, c **B**). The expression of α-SMA in high CCN3 HCC group was higher than that of α-SMA in low CCN3 HCC group (b, **A**). The patients with high expression of CCN3/α-SMA had significantly lower OS and higher CRR than the patients with low expression of CCN3 (a, b, **C**). HCC patients were classified into three subgroups based on CCN3 and α-SMA expression levels, and patients with high expression of CCN3 and α-SMA had the highest CRR and lowest OS (c, **C**)

To illustrate the roles and the relationship of CCN3 and α-SMA in HCC, we used tissue microarrays with 86 clinical samples and found a positive correlation between CCN3 and α-SMA (Pearson 0.238, *p* = 0.027 Fig. 3A*, a*). And the expression of α-SMA in high CCN3 HCC group was higher than α-SMA in low CCN3 HCC group (24.61 ± 8.65 vs. 18.09 ± 11.42, *p* = 0.0014 Fig. 3A, b). The HCC was also evaluated in mRNA level in another 98 clinical samples, and we also evaluated the relationship between CCN3 and α-SMA, and found a positive correlation between CCN3 and α-SMA (Pearson 0.46, *p*<0.001 Fig. 3B).

The expression of CCN3 and α-SMA was also significantly associated with both OS and CRR. The patients in the CCN3-high group had significantly lower OS (*p* = 0.006) and higher CRR (*p*<0.001) than patients in the CCN3-low group. The patients in the α-SMA-high group also had significantly lower OS (*p* = 0.018) and higher CRR (*p* = 0.041) than patients in the CCN3-low group. Next, we classified the patients into three subgroups based on CCN3 and α-SMA expression levels. Group I had low expression of both CCN3 and α-SMA, while Group II had high expression of either CCN3 or α-SMA, and Group III had high expression of both CCN3 and α-SMA. Group I had the best prognosis in the three groups, while OS (*p* = 0.208) and CRR (*p* = 0.126) in Group I was not significantly higher than that of patients in Group II. Also in Group I, the OS (*p* = 0.001) was significantly higher and the CRR (*p* = 0.001) was lower than in Group III. In Group II, the OS (*p* = 0.01) was significantly higher and the CRR (*p* = 0.035) was significantly lower than in Group III (Fig. 3C).

Furthermore, Cox regression analysis revealed a significant association of CCN3-high expression in tumor tissue with tumor dimension (*p* = 0.013), vascular invasion (*p* = 0.023), and tumor encapsulation (*p* = 0.014) of HCC. In regards to α-SMA-high expression in tumor tissue, we found a significant correlation with tumor dimension (*p* = 0.044), and tumor encapsulation (*p* = 0.009) of HCC. No statistically significant association was found with other clinical characteristics (Table. 1). In this section, we proved the expression levels of CCN3 and α-SMA are positively correlated, and the high expression of CCN3 and α-SMA are positively associated with malignant phenotype and poor prognosis in HCC.

### Only when HSCs enter the tumor tissues can they play a promoting role, and CCN3 paracrined by HCC enhances infiltration of HSCs into tumor tissues relating to ERK signaling pathway

**Fig. 4:**
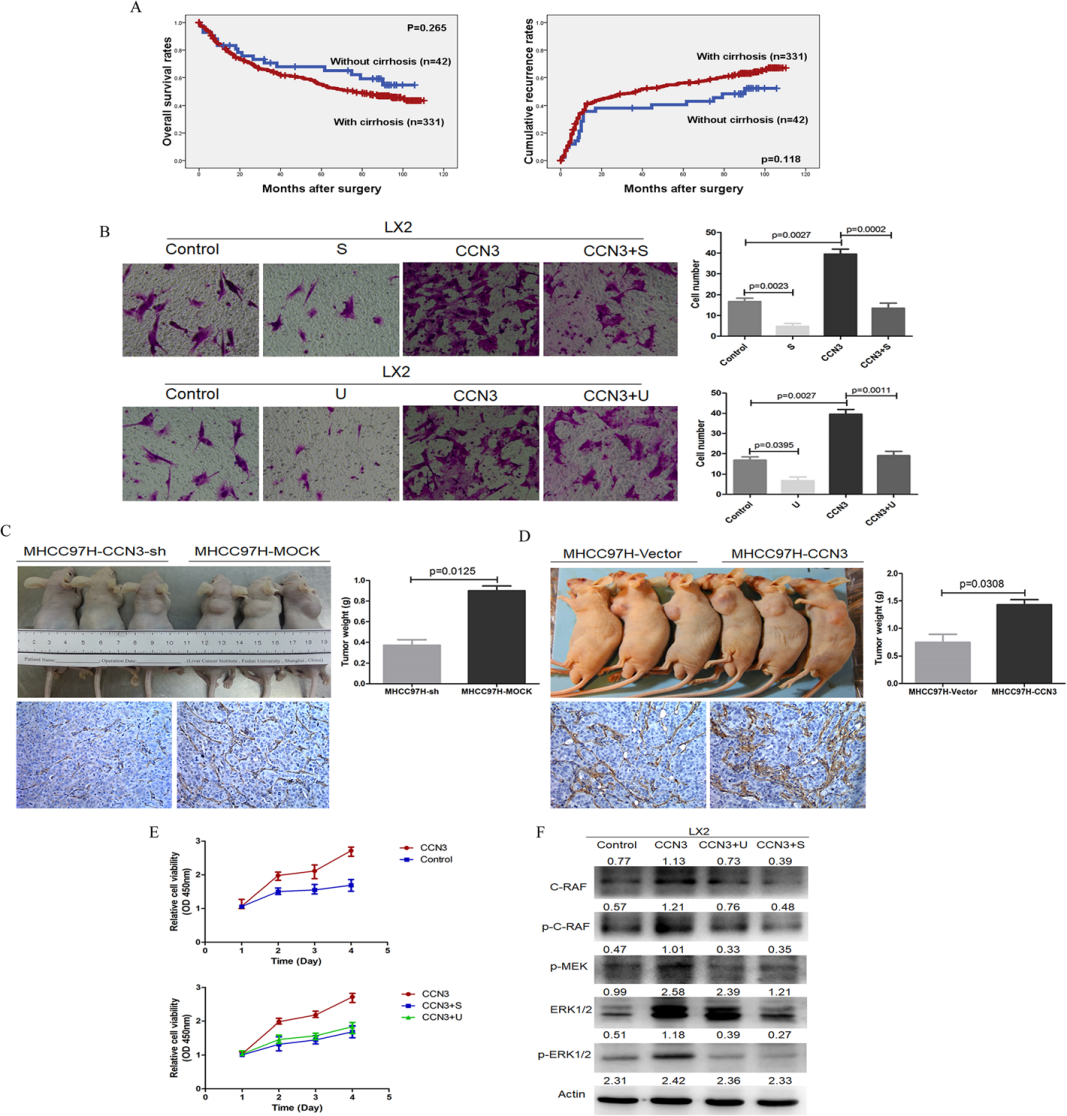
Migration and proliferation ability of HSC is related to CCN3-ERK signaling. There was no significant difference in OS and CRR between patients with or without cirrhosis (**a**). Recombinant CCN3 could significantly enhance the migration and proliferation (**c**) of LX2, which could be reversed by S (sorafenib) or U (U0126) (**b**). The diminished number of HSC and subcutaneous tumor weight were found in mice injected with MHCC97H-CCN3-sh cells in nude mouse models (**d**). The increased number of HSC and the enhanced subcutaneous tumor growth were found in the MHCC97H-CCN3 group (**e**). CCN3 could activate ERK signaling pathways with upregulation of RAF, p-RAF, p-MEK and p-ERK, and sorafenib or U0126 showed significant inhibition on ERK signaling (**f**)

We had proven that the number of HSC in HCC tissues was positively correlated with tumor dimension and OS in HCC patients. Although peripheral liver tissue with or without cirrhosis can partially affect the OS and CRR of HCC patients, there was no significant difference in OS (*p* = 0.265) and CRR (*p* = 0.118, Fig. 4a). Therefore, we concluded that only when HSC cells enter the tumor tissues can they play a promoting role in cancer.

To investigate the role of CCN3 in modulating the transmigration of HSC, we treated LX2 cells with recombinant CCN3, which could significantly enhance the migration (16.75 ± 3.30 vs. 39.50 ± 4.79, *p* = 0.0027, Fig. 4b). In nude mouse models, the diminished number of HSC with low expression of α-SMA was found in mice injected with MHCC97H-CCN3-sh cells, meanwhile, diminished subcutaneous tumor weight in nude mouse models was found in the MHCC97H-CCN3-sh group (0.91 ± 0.19 vs. 0.37 ± 0.11 *p* = 0.0125, Fig. 4c). The overexpression of CCN3 through lentiviral infection of MHCC97H cells in mice led to increased number of HSC with high expression of α-SMA, and the enhanced subcutaneous tumor growth was found in the MHCC97H-CCN3 group (0.74 ± 0.30 g vs. 1.43 ± 0.19 g *p* = 0.0308, Fig. 4d).

Further, we investigate the associated mechanism of CCN3 on LX2, and proved CCN3 led to the activated ERK signaling pathways with upregulation of RAF, p-RAF, p-MEK and p-ERK. On the other hand, LX2 cells treated with sorafenib (2 μmol/L) or MEK1/2 inhibitor U0126 (10 μmol/L) showed significant inhibition on ERK signaling (Fig. 4f). To investigate the role of CCN3 and ERK signaling pathways on the migration and proliferation of LX2, we treated LX2 cells with sorafenib (2 μmol/L) and MEK1/2 inhibitor U0126 (10 μmol/L) again. Sorafenib treatment could significantly inhibit the migration (16.75 ± 3.30 vs. 4.75 ± 2.76, *p* = 0.0023) and proliferation of LX2. To further clarify the role of ERK signaling activated by CCN3 in the migration of LX2, Sorafenib or U0126 combined with CCN3 exhibited decreased migration (39.50 ± 3.30 vs. 13.51 ± 4.93, *p* = 0.0002; 39.50 ± 3.30 vs. 19.01 ± 4.32, *p* = 0.0011) and proliferation of LX2 with down-regulated ERK signaling (Fig. 4b, e*).* In this section, we proved only when HSC cells enter the tumor tissues can they play a promoting role in cancer, and enhanced migration and proliferation of HSC were relating to ERK signaling pathway after treated by CCN3.

### CCN3 induce the remodeling of HSC with elevation of cytokines paracrine relating to HCC malignancy

**Fig. 5:**
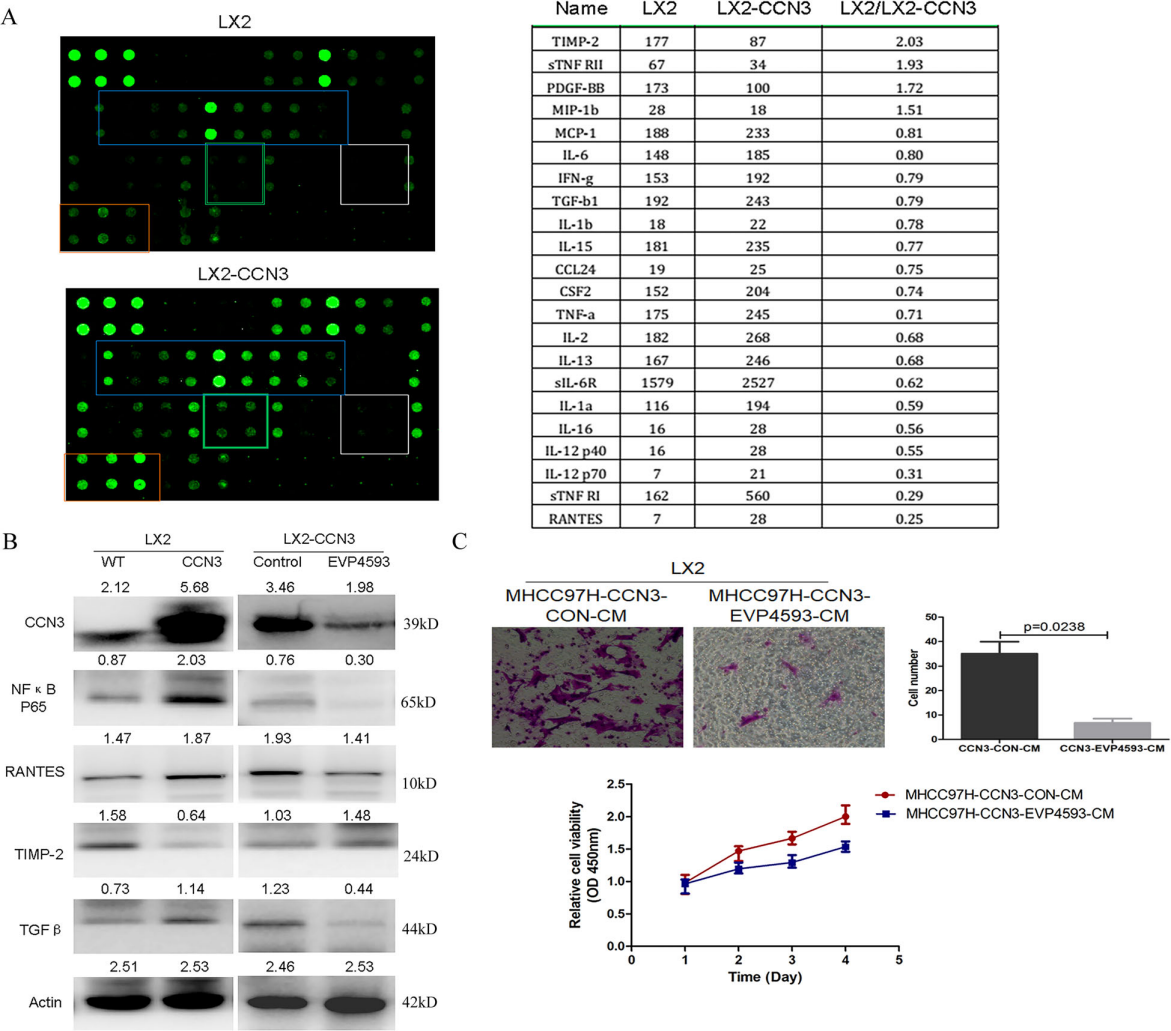
CCN3 induce the remodeling of HSC with elevation of cytokines relating to HCC malignancy. The significantly changed cytokines expression profiles were found in CCN3 treated LX2 by cytokines array (**a**). RANTES, TGFβ and TIMP-2 were selected for immunoblotting, and NFκB was proved as one of the control signaling pathway (**b**). The reduced migration and inhibited proliferation of LX2 were proved in the CM from NFκB inhibitor EVP4593 treated MHCC97H (**c**)

While studying the direct role of CCN3 on HSC, we treated LX2 with CCN3 and found the significantly up-regulated cytokines expression profiles by cytokines array in LX2-CCN3, with the up-regulated cytokines of RANTES, IL-16, IL-1a, IL-13, IL-2, TNFa, TGFβ, and MCP-1 et al., and down-regulated cytokines of TIMP-1, sTNFRII et al. (Fig. 5a). Further, RANTES and TGFβ were selected for immunoblotting and we proved the significant increase of the two cytokines, which were regulated by NFκB signaling after we overexpressed CCN3 in LX2. To validate this effect, NFκB signaling was inhibited with concomitant down-regulation of RANTES, TGFβ, and up-regulation of TIMP-2 after treatment with NFκB inhibitor EVP4593 (Fig. 5b). To investigate the effect of NFκB signaling pathways of HCC on the migration and proliferation of HSC, we treated HCC cells with NFκB inhibitor EVP4593, and collected the CM. We proved the reduced migration (35.01 ± 9.89 vs. 6.75 ± 3.50 *p* = 0.0238) and inhibited proliferation (1.67 ± 0.75 vs. 1.25 ± 0.08 *p* = 0.0016) of LX2 in the CM from EVP4593 treated HCC (Fig. 5c). In this section, we proved CCN3 induce the remodeling of HSC with elevation of cytokines relating to HCC malignancy.

## Discussion

Approximately 90% of HCC develops in chronically damaged tissue due to liver cirrhosis, and chronic hepatitis B virus infection remains the major risk factor [9]. Cirrhosis is closely affecting the liver function and is strongly associated with the development of HCC [10]. This milieu of fibrosis further reduces the responsiveness of tumor cells towards various clinical treatments, thus directly affecting the tumor malignancy progression [11]. It is believed that focusing upon cirrhosis makes it possible to evaluate HCC heterogeneity and explore new therapies to move towards a more personalized medical approach.

A previous study has demonstrated that HSCs play a key role in causing liver cirrhosis and infiltration into the HCC milieu [12]. HSCs are well known to be one of the key cell types that contribute to the pathogenesis of cirrhosis, which are activated in response to liver damage and trans-differentiate into mesenchymal fibroblasts (MFs), and play a key role in aggravating hepatic fibrosis to the form of waterfall effect [13]. It is reported that peripheral liver tissue with cirrhosis can affect the progression of HCC patients [14], while in the present study, we proved there was no significant effect of cirrhosis on OS and CRR of HCC patients, and we speculate HSCs transmigrating into HCC tissue are the key to promote HCC malignant progression. It is known that HSCs can infiltrate into the stroma of liver tumors, where they are remodeled as cancer associated fibroblasts (CAFs) and play a critical role in HCC progression [11]. To explore the mechanism of oxaliplatin resistance from the perspective of HCC microenvironment, in the present study, we proved increased accumulation of HSCs with fibrous connective tissue and collagen in oxaliplatin-resistant HCC tissue milieu. In HCC patients, we also proved the expression of α-SMA representing the number of HSCs was significantly associated with malignant phenotype, and the patients with high α-SMA expression in HCC tissue had poor prognosis. Previously, we constructed oxaliplatin-resistant HCC models and found the CCN family was significantly changed in oxaliplatin-resistant HCC. And the paracrine of CCN3 from HCC was one of the significantly up-regulated protein in HCC, especially in oxaliplatin-resistant HCC [5].

The CCN family, first described by P. Bork in 1993, is a small, six-member family of cysteine-rich regulatory proteins found in humans. Members of this secreted protein family comprise a secretory signal peptide followed by four structural domains; namely, insulin-like growth factor binding proteins (IGFBP), von Willebrand factor type C repeat (VWC), thrombospondin type I repeat (TSP-1), and carboxy-terminal domain (CT) [3]. Because the four unique globular modules share homology with functional domains of various extracellular proteins, CCN proteins have emerged as localized multitasking signal integrators in the inflammatory microenvironment [15]. Many studies have shown how physiological and pathological events acting on each catalytic domain of CCNs affect differentiation [16], adhesion [17], migration [18], mitogenesis [19], chemotaxis [20] and angiogenesis [21]. Recently, interest in CCN3 has emerged for cancer research because of the protein’s central roles in cell regulation [3, 22]. Chen [23, 24] et al. has shown that prostate cancer-derived CCN3 can induce M2 macrophage infiltration, relating to the construction of the prospective environment conducive to prostate cancer metastasis. Previously, we had reported that HSCs could interact with hepatoma cells via secretion of cytokines, which play a critical role in modulating the malignant phenotypic changes of HCC [6, 25]. In the present study, we proved the infiltration of HSCs into the HCC milieu relating to CCN3 paracrine of HCC, and CCN3 could elevate the cytokines paracrined by HSCs relating to enhanced HCC malignancy, all of which indicate the important role of CCN3 in the crosstalk between HCC and HSCs.

Cancer cells do not manifest the disease alone and the stroma is inappropriately activated in cancer to contribute to malignant characteristics of tumor cells. The stroma and the tumor cells always create a complex system with reciprocal signaling [26]. Uncontrolled or sustained damage of liver tissue with the activation and infiltration HSC is now recognized as a hallmark feature of HCC development and metastasis [27]. Accumulating evidence supports the concept that activated HSCs are the main matrix-producing cells in the process of liver fibrosis, resulting in ECM accumulation and HCC progression [28, 29]. The functions of CCN family proteins are involved in the regulations of HCC microenvironment [30]. It has been reported the enhanced expression of CCN3 was found in HCC samples when compared to levels in matched non-cancerous tissues, and these results suggest that CCN3 was associated with the development of tumors [31, 32]. In the present study, we proved the expression of CCN3 is up-regulated in oxaliplatin-resistant HCC, which induced the remodeling of HSCs with elevation of cytokines such as RANTES, IL-16, IL-1a, IL-13, IL-2, TNFa, TGFβ, and MCP-1 et al., resulting in the maintenance of oxaliplatin-resistance of HCC. This may in part reflect the abnormal HCC microenvironment, which acts to support the growth and chemoresistance of HCC.

Currently, drug development in HCC remains focusing on HCC itself, which could be ignorance of the importance of the HCC microenvironment driving a tumor-permissive milieu. Throughout the process of HCC progression, tumor cells constantly communicate with the fibrotic microenvironment and improve their malignant potential. Now we proved that CCN3 is recognized as a hallmark of HCC development and chemo-resistance, and better therapies will hopefully follow the thorough understanding of the biological functions of CCN3 protein.

## Conclusions

From our experimental results and our review of the literature, we propose the following conclusions. (1) The increased infiltration of HSCs and collagen accumulation were found in the microenvironment of oxaliplatin-resistant HCC. (2) CCN3 was one of the significantly increased proteins in the oxaliplatin-resistant HCC, and the levels of CCN3 and α-SMA are positively correlated and positively associated with malignant phenotype and poor prognosis. (3) CCN3 induce the remodeling of HSC with elevation of cytokines paracrine relating to stroma-derived oxaliplatin-resistance in HCC.

## Data Availability

All data generated or analyzed during this study are included in this published article. The datasets used and/or analyzed and materials developed during the current study are available from the corresponding author by reasonable request.

## Abbreviations

AFP: Alpha-fetoprotein
ALT: Alanine aminotransferase
CAFs: Cancer associated fibroblasts
CM: Conditioned medium
CRR: Cumulative recurrence rates
DMEM: Dulbecco’s modified eagle’s medium
HBsAg: Hepatitis B surface antigen
HBV: Hepatitis B virus
HCC: Hepatocellular carcinoma
HSCs: Hepatic stellate cells
NOV: Nephroblastoma overexpressed proteins
OPN: Osteopontin
OS: Overall survival rates
PCR: Polymerase chain reaction
TF: Tissue factor
